# Characterization of the porcine nutrient and taste receptor gene repertoire in domestic and wild populations across the globe

**DOI:** 10.1186/1471-2164-15-1057

**Published:** 2014-12-03

**Authors:** Elizabete C da Silva, Nadia de Jager, William Burgos-Paz, Antonio Reverter, Miguel Perez-Enciso, Eugeni Roura

**Affiliations:** Centre for Research in Agricultural Genomics (CRAG), CSIC-IRTA-UAB-UB, 08193 Bellaterra, Spain; Faculty of Agronomy and Veterinary Medicine, Darcy Ribeiro UnB, Brasília-Asa Norte, Distrito Federal, 70.910-970 Brazil; Centre for Nutrition and Food Sciences, The University of Queensland, St. Lucia, 4067 Australia; Commonwealth Scientific and Industrial Research Organisation (CSIRO), Agriculture Flagship, Queensland Bioscience Precinct, 306 Carmody Road, St. Lucia, QLD 4067 Australia; Institut Català de Recerca i Estudis Avançats (ICREA), Carrer de Lluís Companys 23, Barcelona, 08010 Spain

**Keywords:** Pig, Nutrition, GPCR, Taste receptor, Bitter, T1R, T2R, SNPs, Population genomics

## Abstract

**Background:**

The oral GPCR nutrient/taste receptor gene repertoire consists of the *Tas1r* family (sweet and umami tastes), the *Tas2r* family (bitter taste) as well as several other potential candidate sensors of amino acids, peptones and fatty acids. Taste/nutrient receptors play a fundamental role in survival through the identification of dietary nutrients or potentially toxic compounds. In humans and rodents some variations in taste sensitivity have been related to receptor polymorphisms. Some allelic variants, in turn, have been linked to the adaptation to specific geographical locations and dietary regimes. In contrast, the porcine taste/nutrient receptor repertoire has been only partially characterized and limited information on genetic variation across breeds and geographical location exists. The present study aims at filling this void which in turn will form the bases for future improvements in pig nutrition.

**Results:**

Our results show that the pig oral repertoire of taste/nutrient receptors consists of at least 28 receptor genes with significant transcription measured for 27. When compared to humans and rodents, the porcine gene sequences encoding sensors for carbohydrates, amino acids and fatty acids were highly conserved whilst the bitter taste gene family (known as *Tas2rs*) showed high divergence. We identified 15 porcine *Tas2rs* of which 13 are orthologous to human sequences. The single nucleotide polymorphism (SNP) sequence analysis using 79 pig genomes, representing 14 different breeds/populations, revealed that the *Tas2r* subset had higher variability (average π =2.8 × 10^-3^) than for non-bitter taste genes (π =1.2–1.5 × 10^-3^). In addition, our results show that the difference in nutrient receptor genes between Asian and European breeds accounts for only a small part of the variability, which is in contrast with previous findings involving genome wide data.

**Conclusions:**

We have defined twenty-eight oral nutrient sensing related genes for the pig. The homology with the human repertoire is high for the porcine non-bitter taste gene repertoire and low for the porcine *Tas2r* repertoire. Our data suggests that bitter taste is a plastic trait, possibly associated with the ability of pigs to adapt to diverse environments and that may be subject to balancing selection.

**Electronic supplementary material:**

The online version of this article (doi:10.1186/1471-2164-15-1057) contains supplementary material, which is available to authorized users.

## Background

The pig, *Sus scrofa*, appeared in South East Asia ~4.2 million years ago (M) [1], colonizing a wide range of habitats thereafter including Europe and North Africa. European and Asian wild boars are estimated to have diverged ~1.2 M [2]. The wild boar is among the first of the domesticated livestock species, an event that occurred approximately 8,000-10,000 BC both in Europe and in Asia in independent events [3, 4]. Today, thanks to the intense modern breeding and selection programmes, the pig is one of the most economically important domestic species worldwide providing a relatively cheap source of dietary protein for humans. The species *Sus scrofa* is highly variable at both the DNA and phenotypic levels and there are 200-300 pig breeds currently recognized [5, 6]. Consequently, the study of pig diversity from different ecosystems and breeds including wild and domestic populations may uncover phenotype-genotype relationships of high evolutionary and adaptive physiology relevance. In particular, dietary adaptation through taste sensory mechanisms is emerging as a major evolutionary selection pressure [7, 8]. Taste receptors (hereinafter referred to as TRs) and their genes (*Tasrs*, nomenclature consistent with the review by Bachmanov and Beauchamp [9]) are known to monitor the presence of dietary compounds in the oral cavity. With the exception of the salty and sour tastes, all other candidate receptors known to date related to taste and nutrient sensing belong to the family of G-protein coupled receptors (GPCRs). Salty and sour perceptions seem to be related to ligand gated transmembrane channels. More specifically, these channels consist of tetrameric epithelial sodium channels (involving three genes *ENaC*_*α,β,γ*_) for salty; and dimeric hydrogen gated channels (involving two genes *PKD1L3* and *PKD2L1*) for sour [9]. Both multimeric transmembrane channels are quite ubiquitous and do not seem to be specific to sensory cells, hence have not been included in this study. On the other hand, the taste system includes two main families of GPCRs. Family 1 is related to simple sugars and some L-amino acids present in the diet (hereinafter referred to as Tas1rs). Family 2 is part of the sensory mechanism to identify potentially toxic compounds and elicits bitter taste (hereinafter referred to as Tas2rs) [9]. Other GPCRs have been related to nutrient sensing in the oral cavity and include the sensing of amino acids and peptones (mGluR1, mGluR4, GPRC6A, CaSR and GPR92), medium and long chained saturated and unsaturated fatty acids (GPR40, GPR41, GPR43, GPR84 and GPR120) [9, 10]. Overall, the oral chemosensory gene repertoire can be potentially divided into those receptors identifying nutrients (e.g. sugars, amino acids and fatty acids) which in turn would elicit a positive hedonic sensation, and receptors responding to potential undesirable substances (e.g. plat-derived toxic compounds), which in turn would trigger a repulsive response (bitter).

More precisely, the Tas2r family seems to play a role of particular relevance in species evolution across mammalian species [7]. In a genomic analysis involving 54 vertebrate species (including 41 mammals) Li and Zhang [8] found evidence that the *Tas2r* diversity was associated with the adaptation to the presence of dietary toxins among other selective forces. In addition, genetic selection related to domestication may also be an important driver to dietary adaptations [11]. Thus, we hypothesize that the cluster of *Tas2rs* across pig breeds from different geographical origins and/or selection pressure (such as the one observed in commercial breeds) will show a higher presence of polymorphisms than the non-bitter nutrient/taste sensing genes.

The genome of the Duroc breed of swine was sequenced by the International Swine Genome Sequencing Consortium (SGSC) and the information was made publically available in 2010 [12]. In 2013 a reviewed annotation was released which identified part of the porcine taste receptor repertoire [2]. A total of 25,322 genes (including 566 pseudo genes) are currently annotated in the *Sus scrofa* assembly 10.2 (Ensembl database v. 75). However, the nutrient sensing and taste receptor gene repertoire in pigs has only been partially described [2, 13, 14] and their diversity across the *Sus scrofa* population remains unknown.

The objective of our study is to update the current porcine genome annotation regarding nutrient sensors or taste receptors and study their diversity. Here we quantify and compare the variability in nutrient and taste receptor genes across different domestic breeds and wild boars spread around the world. Given the potential role of bitter perception in environmental adaptations, we will test the hypothesis that the *Tas2r* repertoire in pigs has a higher diversity than the non-bitter taste receptors.

## Results

### Prediction of the porcine taste and nutrient receptor gene repertoire

**Figure 1:**
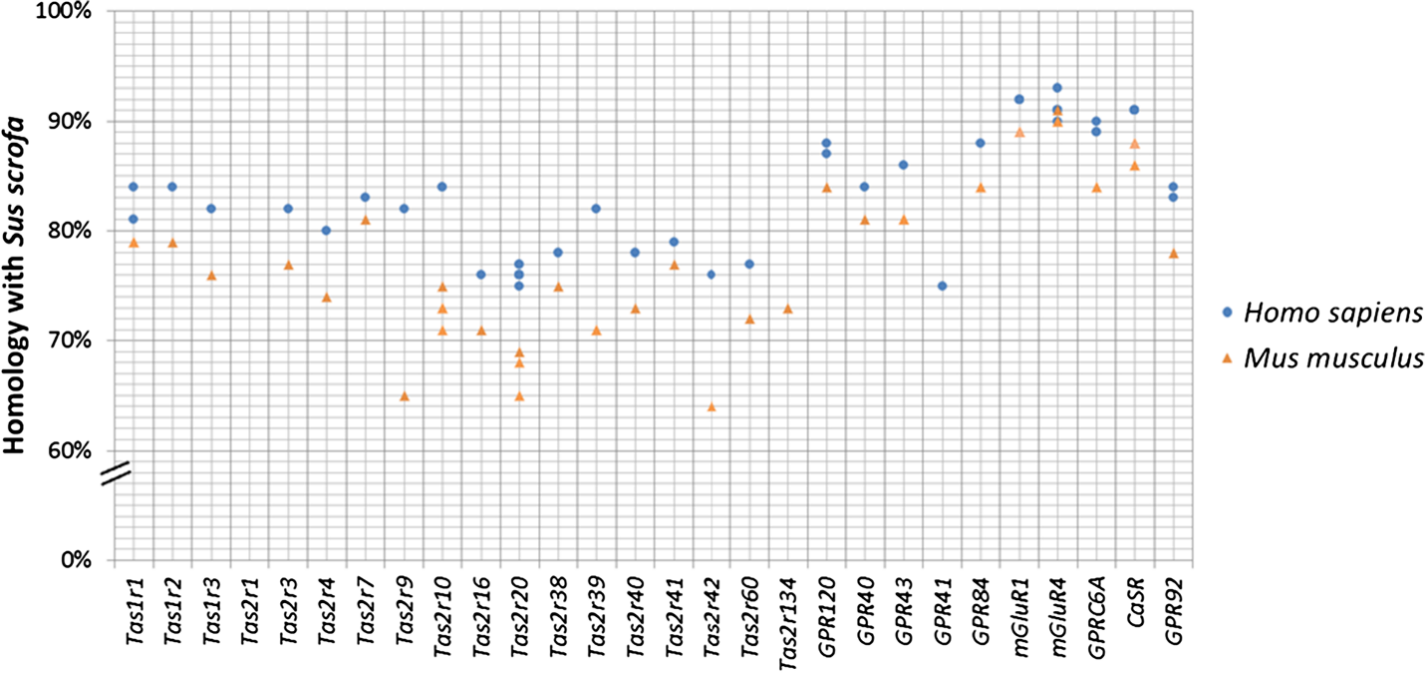
**BLAST results showing percent homology.** Shown is the percent identity between the porcine nutrient sensor and taste receptor gene (*Tasr*) mRNA sequences from the pig genome refseq database (*Sus scrofa* 10.2) blasted to the human (●) and mouse (▲) genomes. In total there were 28 nutrient sensor and *Tasr* mRNA sequences identified in the pig genome as being orthologous to respective taste receptor genes in human and mouse. Included are 3 *Tas1rs*, 15 *Tas2rs*, 5 fat/fatty acid genes, 3 amino acid genes, 1 calcium receptor gene and 1 peptone receptor gene. For those genes that had more than one blast hit (see Additional file 1), with different homology percentages, this can be seen as more than one ●/▲ on the graph corresponding to that gene. In the case where the hits had the same homology percentage, the symbols overlap.

In order to identify the *Tasr* repertoire in the porcine genome, we carried out BLAST searches using known human (n =37) and mouse (n =47) mRNA sequences. We excluded putative sour and salty taste receptor genes from the analysis because of their multimeric nature, ubiquitous expression (i.e. not unique to taste sensory cells) and not being GPCRs. The genes were grouped based on nutrient sensing: sugars (*Tas1r2* and *Tas1r3*); amino acids and peptones (*Tas1r1*, *Tas1r3*, *mGluR1*, *mGluR4*, *GPRC6A*, *CaSR* and *GPR92*); fatty acids (*GPR40*, *GPR41*, *GPR43*, *GPR84* and *GPR120*); and bitter compounds (the *Tas2r* sub-family). Figure 1 shows the homology percentage between the known *TASRs* and *Tasrs* in human and mouse, respectively compared to those found to match in the pig genome. We have included 15 *Tas2r*s, for which the current annotation denotes 11 of these to be protein coding and 4 to be pseudo genes (Additional file 1). The porcine *Tas2r* repertoire appears to differ significantly from human and mouse repertoires. For example, seven human *TAS2R* (numbers 14, 19, 20 31, 43, 45, 46 and 50) and three mouse *Tas2r* (numbers 120, 123 and 117) show high homology (≥65%) to only a single pig bitter receptor pseudo gene, *Tas2r20*. In contrast, the pig *Tas2r1* and *Tas2r134* have no human orthologs.

Of all the 28 porcine genes studied, the cluster of genes sensing amino acids showed the highest homologies to their human orthologs ranging from 90% to 93% (Additional file 1). The peptone receptor, *GPR92*, had 84% homology with its human ortholog. With the exception of the *GPR41* (75% homology), the fatty acid receptors and the three *Tas1rs* showed medium to high identities between the two species ranging from 82% to 88%. Finally, the lowest homologies identified between pig and human *Tasrs* were amongst the porcine *Tas2r* family and *GPR41*. In addition, when comparing the pig *Tasr* repertoire to the mouse, the gene homologies follow a similar pattern.

### Expression of the porcine GPCR nutrient sensor and taste receptor gene repertoire in circumvallate papillae

**Figure 2:**
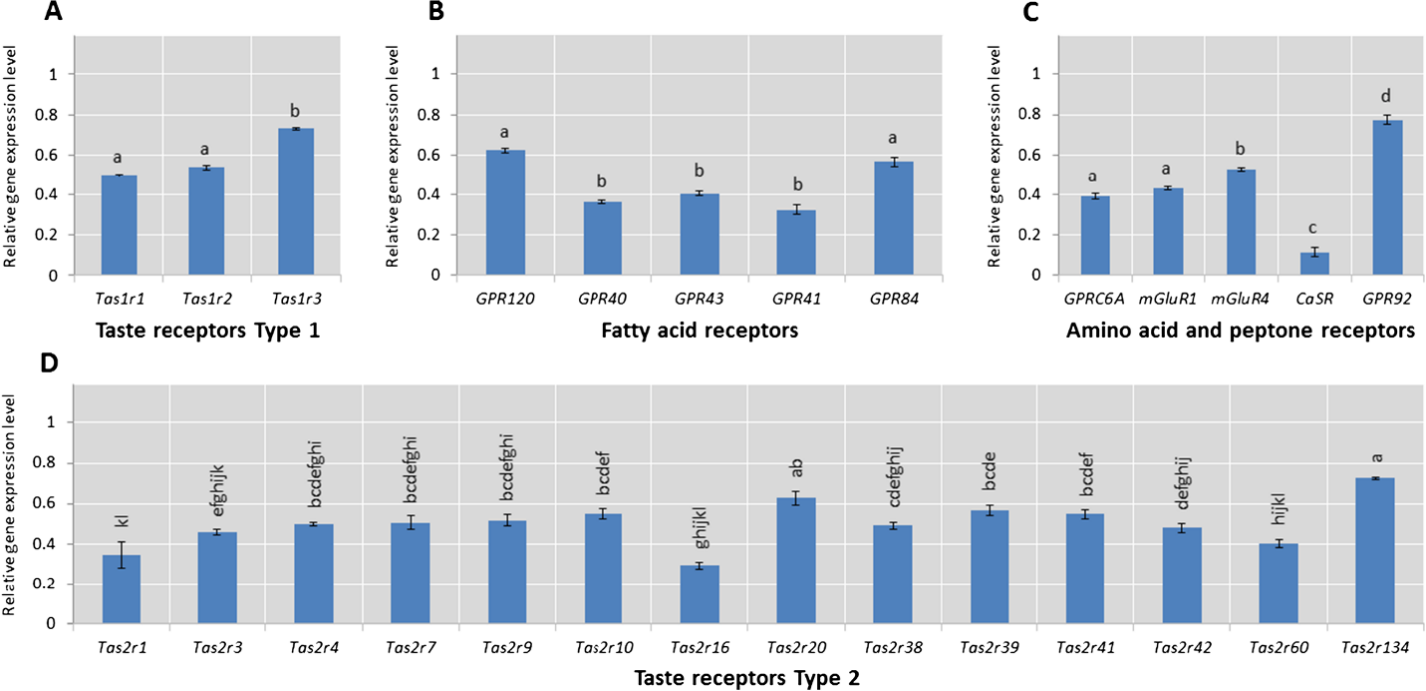
**Normalized gene expression levels for taste receptor/nutrient sensor genes in circumvallate papillae of pigs.** Each bar represents the average gene expression level for 6 biological replicates and the error bars represent the standard error of the mean. The different categories of genes are **(A)** Taste receptors type 1, **(B)** Fatty acid receptors, **(C)** Amino acid and peptone receptors and **(D)** Bitter taste receptors or Taste receptors Type 2. The normalisation was performed relative to two reference genes *RPLP* and *ACTB* and the expression level is provided as a fold change compared to the overall average expression level of *Tas1r1* in circumvallate papillae. The letters of the alphabet denote statistical significance, where the same letter refers to no significance (P>0.05) and where a different letter refers to significant differences (P<0.05).

We determined whether the candidate porcine *Tasrs* were transcribed into mRNA in tongue circumvallate papillae using real time PCR. Following standard procedures, total RNA was extracted from porcine papillae and reversed transcribed into cDNA before carrying out the PCR assays (see methods). Figure 2 shows the relative *in vivo* gene expression levels of all the genes identified in our study as constituting the pig *Tasr* repertoire in pig circumvallate papillae. All *Tasrs* identified were significantly expressed, with the exception of *Tas2r40* which was not measured; due to it not satisfying our criteria of being a protein coding *Tas2r* (refer to discussion). The results showed that *GPR92* and *Tas2r134* had the highest and *CaSR* the lowest relative gene expression levels. Of the *Tas1r* subfamily, *Tas1r3* is expressed significantly (P <0.01) higher than *Tas1r1* and *Tas1r2*. Amongst the *Tas2r* repertoire, we observed the highest expression levels for *Tas2r20* and *Tas2r134*. In contrast, *Tas2r1*, *Tas2r16* and *Tas2r60* were found to have a relatively low gene expression level. Two of the fatty acid sensors had higher (P <0.01) expression levels (*GPR120* and *GPR84*) compared to the other three (*GPR40*, *GPR41* and *GPR43*). Among the group of genes with specificity to amino acid sensing, *CaSR* showed a significantly (P < 0.01) lower abundance than the rest.

### Species wide variant discovery

Out of all 28 gustatory genes identified, we carried out a comprehensive variability analysis of the sequence of 21 genes that were present in the current porcine assembly (build 10.2). The seven genes excluded from the analysis were *Tas1r2*, *Tas2r1*, *Tas2r134*, *Tas2r3*, *Tas2r40*, *Tas2r4* and *GPR92,* because they were either not annotated or annotated in contigs and not in any of the 18 porcine autosomes or sex chromosomes.

Using high throughput sequencing data from 79 samples distributed worldwide (methods, Table 1), a total of 12,235 SNPs were found across all 21 genes and 10 kb flanking regions (Table 2). The average rate of transitions vs. transversions was t_i_/t_v_ =2.35, similar to the genome wide rate in pigs [15] and similar to that found in other mammalian species [16, 17]. A total of 8,962SNPs (73%) had been previously assigned reference SNP numbers available in the Single Nucleotide Polymorphism Database (dbSNP v. 138). Out of 8,259 SNPs positioned between 5’ and 3’ UTRs, 7,963 were in introns, 296 in exons in the protein coding regions of the genes, and only 17 in UTRs (12 in 5’UTR and 5 in 3’UTR). Among functional coding SNPs, one stop lost, one stop gained, 167 synonymous and 110 non-synonymous mutations were found. Additional file 2 contains all SNPs with reference SNP ID number or rs ID if available, reference and alternative allele, amino acid change; SIFT score for non-synonymous changes, and frequency of each variant, globally and by population. The 3,274 novel SNPs have been reported to dbSNP (reference ss1432164463).

**Table 1:** **The Groups of pig breeds included in this study**

Group	Breeds	Country	Code	N (n)
International (INT)	Large White	NA	LW	15 (1)
	Landrace	Several countries	LR	5
	Duroc	Several countries	DU	4
	Pietrain	Several countries	PI	5
	Hampshire	Several countries	HS	2
European domestic	Iberian	Spain	IB	4 (4)
European wild boar	wild boar	Netherlands, Switzerland, France and Spain	EUWB	9 (2)
Asian domestic	Meishan, Xiang, Jiangquhai and Wuzhishan	China	ASD	8
Asian wild boar	Wild boar	Japan, South and North China, East Russia	ASWB	6 (1)
Creole	Creole, Ossabaw, Yucatan	Peru, Cuba, Argentine and United State	CR	14 (13)
Brazilian	Brazilian^a^	Brazil	BR	3 (3)
No group	Tamworth	United Kingdom	TW	1 (1)
No group	‘Manchado Jabugo’	Spain	MJ	1 (1)
Outgroup	Sumatra’s wild boar	Sumatra	SWB	2

Provided are the details on geographical distribution, code and number of individual genomes (N) of the 79 pigs analyzed.

^a^Piau, Monteiro and Moura breeds, n = Quantify of new samples sequenced in this study.

**Table 2:** **General information about the pig taste receptor genes used in this study**

				Total region		Gene region			SNP distribution by class and functional consequences								
Gene group	Genes	exon*	Chr.	Coordinates (bp)	SNPs	Coordinates (bp)	% miss	SNPs	Intergenic	Exon	Intron	5’UTR	3’UTR	Stop lost	Stop gained	Sy	Ns
Bitter	*Tas2r20*	1	5	63894140-63915054	310	63904140-63905054	10.98	13	297	13	-	-	-	-	-	5	8
	*Tas2r9*	1	5	63971739-63982674	229	63976739-63977674	9.10	6	223	6	-	-	-	-	-	0	6
	*Tas2r10*	1	5	63958146-63971725	266	63965446-63966375	12.12	9	257	9	-	-	-	-	-	2	7
	*Tas2r42*	1	5	63857091-63878041	257	63867091-63868041	10.06	17	240	17	-	-	-	-	-	9	8
	*Tas2r16*	1	18	25873452-25894354	474	25883452-25884354	10.25	16	458	16	-	-	-	-	-	8	8
	*Tas2r38*	1	18	8347518-8368525	79	8357518-8358525	16.13	21	58	21	-	-	-	-	-	11	10
	*Tas2r39*	1	18	7348848-7369855	310	7358848-7359855	10.08	28	282	28	-	-	-	-	1	8	19
	*Tas2r41*	1	18	7008806-7029729	88	7018806-7019729	20.90	18	70	18	-	-	-	-	-	6	12
	*Tas2r60*	1	18	7035247-7056597	110	7045247-7046597	11.59	24	86	14	10	-	-	-	-	10	4
	*Tas2r7*	1	5	63982692-63996080	254	63985142-63986080	8.82	10	244	10	-	-	-	-	-	5	5
Aminoacid	*Tas1r3* ^a^	6	6	58109541-58116535	34	58111612-58115907	57.76	17	17	14	3	1	4	-	-	8	1
	*mGluR4*	10	7	34829241-34938071	2344	34839241-34928071	40.00	1976	368	36	1940	8	-	-	-	26	2
	*GPRC6A*	3	1	50121244-50146085	253	50131244-50136085	11.74	41	212	6	35	-	-	-	-	3	3
	*mGluR1*	7	1	21466379-21824964	5671	21476379-21814964	16.07	5421	250	20	5401	3	1	-	-	15	1
	*CaSR* ^a^	6	13	147897932-147940283	345	147907932-147935070	75.62	138	208	22	116	-	-	-	-	20	2
	*Tas1r1*	7	6	62349877-62363714	117	62350603-62363204	31.62	112	5	13	99	-	-	1	-	7	5
Fatty acids	*GPR40*	1	6	40335081-40343065	89	40339566-40340468	50.96	8	81	8	-	-	-	-	-	4	4
	*GPR43*	1	6	40272039-40293031	243	40282039-40283031	40.38	11	232	11	-	-	-	-	-	10	1
	*GPR41*	1	6	40323922-40335070	172	40333922-40334902	43.46	11	161	11	-	-	-	-	-	8	3
	*GPR120*	3	14	114733575-114769008	516	114743575-114765158	18.73	360	156	1	359	-	-	-	-	1	0
	*GPR84*	2	*5*	20404644-20416116	74	20410225-20411195	31.99	2	72	2	-	-	-	-	-	1	1
	Total			778077†	12235	480139†		8259	3977	296	7963	12	5	1	1	167	110

Details included are the chromosome, start and end position, total SNPs and their distribution by genomic region and functional consequence, and missing data (%miss). This analysis was performed in 77 pigs (the two Sumatran wild boars are not included).

^a^Genes excluded because of high missing rate, Sy = Synonymous and Ns = Nonsynonymous sites. *Number of exons per genes.. † Total length in base pairs for all genes (bp).

The % of missing data, for every position, was computed as the number of non-callable genotypes, either because of low quality or low depth (a minimum depth of 5× was required), divided by total number of samples (77). Total missing rate was the average across positions.

We used Sorting Intolerant From Tolerant (SIFT) tool [18], as implemented in Ensembl Variant Effect Predictor, to predict amino acid changes that may affect protein function of nsSNPs subset from dbSNP (http://www.ncbi.nlm.nih.gov/SNP/) [19]. While these *in silico* tools are not always reliable, they do provide guidelines as to what SNPs to prioritize in follow up functional studies. Out of 110 nsSNPs for the investigated *Tasrs*, it was possible to predict tolerance index for 59 SNPs, of which 11 (rs320709106, rs342189509, rs342228000, rs345262132, rs339482728, rs325274060, rs330666697, rs323728911, rs318787211 from dbSNPs; and 5:63977077 and 1:21476805 from novel SNPs) presented a tolerance index score below 0.05, and can therefore be considered potentially deleterious to protein function (Additional file 2). In general, and in agreement with the potentially deleterious nature of these mutations, these alleles were rare and mostly present in a single population; they are probably recent mutations that have not been purged yet. However, a few interesting exceptions exist. For instance, nsSNP rs330666697 (*Tas1r1*) was at intermediate frequency in Asian domestics (minimum allele frequency MAF =0.43) and is present in international and in American village pigs.

### Patterns of nucleotide variation

A worrying aspect of shotgun Next Generation Sequence (NGS) data is the fact that coverage is a quasi-random process and it is therefore unlikely that all samples have enough depth and quality to be analysed. In our data, we found an average of 20% missing data rate (Table 2), which makes it necessary to use methods that account for this. The missing rate was as high as 50% for two genes, *CaSR* and *Tas1r3*, and these were removed from further analyses. Consequently, for the rest of the work we discuss the results relevant only to the 19 loci remaining.

Nucleotide diversity per nucleotide and global fixation indices (F_ST_) were calculated using mstatpop (unpublished, available at http://bioinformatics.cragenomica.es/numgenomics/people/sebas/software/software.html), which provides unbiased estimates of basic population genetic statistics even at high missing rates [18] (Table 3). Species wide, average gene variability was 2.1 × 10^-3^, comparable to that found in the flanking regions (average 1.8 × 10^-3^). Synonymous variability rate (π_s_) was 3.8 × 10^-3^ on average whereas non-synonymous rate (π_a_) was three times lower (average π_a_ =1.21 × 10^-3^), in agreement with most results in the literature [18] and consistent with a prevailing purifying selection model.

**Table 3:** **Nucleotide diversity per gene**

Gene group	Genes	π _t_ ± SE	π _int_ ± SE	π _g_ ± SE	π _e_ ± SE	π _i_ ± SE	π _utr_ ± SE	π _s_	π _a_/ π _s_	F _ST_^a^
Bitter	*Tas2r20*	2.0 ± 0.6	2.1 ± 0.5	2.7 ± 1.2	2.7 ± 1.2	-	-	5.5	0.3359	0.28*
	*Tas2r9*	2.3 ± 0.7	2.4 ± 0.7	1.0 ± 0.6	1.0 ± 0.6	-	-	0.0	NA^†^	0.41*
	*Tas2r10*	1.9 ± 0.6	2.0 ± 0.6	0.8 ± 0.6	0.8 ± 0.6	-	-	1.0	0.6879	0.38*
	*Tas2r42*	2.0 ± 0.6	1.9 ± 0.9	4.7 ± 1.6	4.7 ± 1.6	-	-	10.0	0.3098	0.29*
	*Tas2r16*	3.1 ± 0.8	3.2 ± 0.5	2.2 ± 0.9	2.2 ± 0.9	-	-	4.9	0.2745	0.04
	*Tas2r38*	0.7 ± 0.2	0.5 ± 0.2	3.6 ± 1.3	3.6 ± 1.3	-	-	8.6	0.2371	0.29*
	*Tas2r39*	3.0 ± 0.2	3.0 ± 0.1	4.2 ± 1.5	4.2 ± 1.5	-	-	5.7	0.6438	0.12
	*Tas2r41*	0.5 ± 0.2	0.5 ± 0.2	2.3 ± 1.0	2.3 ± 1.0	-	-	3.3	0.6041	0.29*
	*Tas2r60*	0.8 ± 0.2	0.7 ± 0.2	2.4 ± 0.9	1.6 ± 1.0	4.3 ± 1.6	-	4.8	0.0940	0.38*
	*Tas2r7*	2.9 ± 0.2	3.0 ± 0.2	1.8 ± 0.8	1.8 ± 0.8	-	-	2.8	0.5311	0.15*
Amino acids	*mGluR4*	3.6 ± 0.5	3.2 ± 0.3	3.7 ± 0.4	2.1 ± 0.6	3.7 ± 0.4	0.8 ± 0.7	7.7	0.0188	0.28*
	*GPRC6A*	1.0 ± 0.8	1.1 ± 0.8	0.5 ± 0.2	0.5 ± 0.3	0.6 ± 0.3	-	0.8	0.6738	0.29*
	*mGluR1*	2.1 ± 0.3	1.5 ± 0.2	2.1 ± 0.4	0.8 ± 0.4	2.1 ± 0.7	3.0 ± 1.8	3.5	0.0007	0.18*
	*Tas1R1*	0.8 ± 0.3	0.5 ± 0.8	0.9 ± 0.3	0.4 ± 0.3	1.0 ± 0.3	-	0.8	0.3351	0.36*
Fatty acids	*GPR40*	1.9 ± 0.3	1.9 ± 0.4	1.6 ± 0.8	1.6 ± 0.8	-	-	3.6	0.1940	0.23*
	*GPR43*	1.9 ± 0.6	2.0 ± 0.5	1.5 ± 0.8	1.5 ± 0.8	-	-	5.1	0.0378	0.09
	*GPR41*	2.9 ± 0.6	3.1 ± 0.5	1.1 ± 0.7	1.1 ± 0.7	-	-	3.4	0.0986	0.26*
	*GPR120*	1.9 ± 0.9	1.4 ± 0.6	2.2 ± 0.5	0.3 ± 0.2	2.3 ± 0.7	-	1.3	0.0000	0.15
	*GPR84*	0.5 ± 0.5	0.6 ± 0.5	0.1 ± 0.1	0.1 ± 0.1	-	-	0.1	0.3891	0.26*
Average		1.9 ± 0.6	1.8 ± 0.6	2.1 ± 0.9	1.7 ± 0.9	2.3 ± 0.7	1.9 ± 1.2	3.8		

The nucleotide diversity per kilobase is shown by genomic region (π), synonymous (π_s_) and non-synonymous diversity (π_a_) and fixation index (F_ST_) for each taste receptor gene analyzed in the global pig population excluding the two Sumatran wild boars (n =77).

SE = standard error, ^a^ = F_ST_ computed using total region (intergenic and gene), Nucleotide diversity for total (π_t_), intergenic (π_int_), gene (π_g,_ included: CDS, intron and UTRs), exonic (π_e_), intronic (π_i_) and in UTR region (π_utr_). ^†^Gene without synonymous mutations. *P < 0.05, Significance based on 1000 permutations.

Among gene regions, nucleotide diversity (π_g_) ranged from 0.5 × 10^-3^ in *GPRC6A* to 4.7 × 10^-3^ in *Tas2r42*. Interestingly, bitter taste genes exhibited higher nucleotide diversity on gene regions (average π_g_ =2.6 × 10^-3^) than in intergenic regions (average π_t_ =1.9 × 10^-3^). The opposite was observed in the remaining groups of genes, which showed greater diversity in the complete region, i.e., gene sequence plus 10 kb flanking regions (averages π_t_ =1.9 × 10^-3^ in gene region and 2.1 × 10^-3^ in complete region). Both fatty acid and amino acid receptors showed lower gene nucleotide diversity than bitter taste receptors (Tables 3 and 4). Overall, the gene variability, especially for the bitter taste receptors, are higher than normally reported for the pig species genome wide, which are in the order of 1.2 × 10^-3^ for international pig breeds and 0.7 × 10^-3^ for Iberian pigs [2, 20].

**Table 4:** **Nucleotide diversity per population**

		Bitter		Amino acid		Fatty acid		All ***Tasr***	
Populations*	N	π_t_ ± SE	π_g_ ± SE	π_t_ ± SE	π_g_ ± SE	π_t_ ± SE	π_g_ ± SE	π_t_ ± SE	π_g_ ± SE
International	31	1.8 ± 0.3	2.8 ± 0. 5	1.9 ± 0. 1	1.7 ± 0.0	1.4 ± 0.3	1.0 ± 0.2	1.8 ± 0.3	2.2 ± 0.4
Iberian	04	1.1 ± 0.1	2.0 ± 0. 4	0.5 ± 0.0	0.4 ± 0. 0	0.4 ± 0.0	0.2 ± 0.1	0.7 ± 0.1	1.2 ± 0.2
Creole	14	2.7 ± 0.6	3.3 ± 0.85	1.8 ± 0.3	1.7 ± 0.1	2.5.0 ± 0.3	1.8 ± 0.3	2.5 ± 0.5	2.6 ± 0.5
Brazilian	03	2.8 ± 0.5	3.6 ± 1. 0	2.1 ± 0.0	2.1 ± 0.1	2.9 ± 0.4	1.7 ± 0.3	2.8 ± 0.5	2.90 ± 0.7
Asian	08	2.0 ± 0.3	2.6 ± 0. 7	2.0 ± 0.1	1.7 ± 0.0	2.1 ± 0.4	1.9 ± 0.5	2.1 ± 0.4	2.3 ± 0.6
Asian WB	06	2.5 ± 0.6	2.5 ± 0.60	2.2 ± 0.3	2.1 ± 0.1	1.9 ± 0.2	1.7 ± 0.4	2.4 ± 0.5	2.3 ± 0.6
European WB	09	2.1 ± 0.3	3.1 ± 0. 8	1.0 ± 0.1	0.9 ± 0.0	0.4 ± 0.0	0.5 ± 0.1	1.5 ± 0.2	1.9 ± 0.5
Total	75	2.1 ± 0.4	2.8 ± 0.7	1.7 ± 0.1	1.5 ± 0.1	1.6 ± 0.2	1.2 ± 0.3	2.0 ± 0.3	2.2 ± 0.5

Estimations are per kilobase and were calculated at total (π_t_) and gene region (π_g_) by population and gene group.

Samples from Tamworth, ‘Manchado de Jabugo’, and the two Sumatran wild boars are not included.

*P < 0.05, Significance based on 1000 permutations.

As mentioned, the ratio of non-synonymous to synonymous variants (*ω = π*_*a*_*/π*_*s*_) was smaller than 1 in all genes (Table 3), indicating prevalent purifying selection. Some extreme cases were observed. For instance, we did not find any non-synonymous SNPs in *GPR120* or any synonymous polymorphisms in *Tas2r9*. Four genes (*Tas2r10*, *Tas2r39*, *Tas2r41*, and *GPRC6A*) presented *ω* values higher than 0.5 and smaller than 1, likely due to weak purifying selection (Table 3).

Estimates of nucleotide diversity varied greatly between genes and between populations (Table 4 and Additional file 3). Asian domestic (ASD) and Asian wild boar (ASWB) exhibited a high within-population variability, with average value π_g_ =2.3 × 10^-3^. Iberian population was the least variable (π_g_ =1.2 × 10^-3^), whereas the American village and Brazilian pigs presented the highest levels of diversity π_g_ =2.6 × 10^-3^ and π_g_ =2.9 × 10^-3^, respectively which seem to reflect their admixed ancestry [18]. We also analyzed the nucleotide diversity by gene groups in each population (Table 4). Brazilian, Creole and EUWB population showed the highest variability for *Tas2rs*, mainly when we analyzed only the gene regions (averages π_g_ =3.6 × 10^-3^ ± 1.0 × 10^-3^, π_g_ =3.3 × 10^-3^ ± 0.8 × 10^-3^ and π_g_ =3.1 × 10^-3^ ± 0.8 × 10^-3^, respectively). Iberian population, in comparison to other populations, showed almost two times lower nucleotide diversity for this same gene group (average π_g_ =2.0 × 10^-3^ ± 0.4 × 10^-3^, Table 4). Therefore, except in the Iberian pig where we analyzed the highly inbred strain ‘Guadyerbas’ [21], the rest of porcine populations analyzed exhibit considerable variability in these genes.

### Structure and Phylogeography

Like most domestic species, the pig is arranged in breeds with specific phenotypic differences that are genetically isolated or with limited genetic interchange. High differentiation indices (F_ST_) are therefore expected in such a structured species with a wide range of distribution and many specialized breeds which prevent the gene flow between them. Not unexpectedly, the global estimate of the F_ST_ over all populations per each gene was significantly different from zero, except in *Tas2r16*, *Tas2r39*, *GPR43* and *GPR120* (Table 3) indicating a widespread population differentiation and limited gene flow between populations. By groups, the fatty acid receptors had the lowest degree of differentiation. Significant F_ST_’s ranged from 0.15 to 0.41 (Table 3).

**Figure 3:**
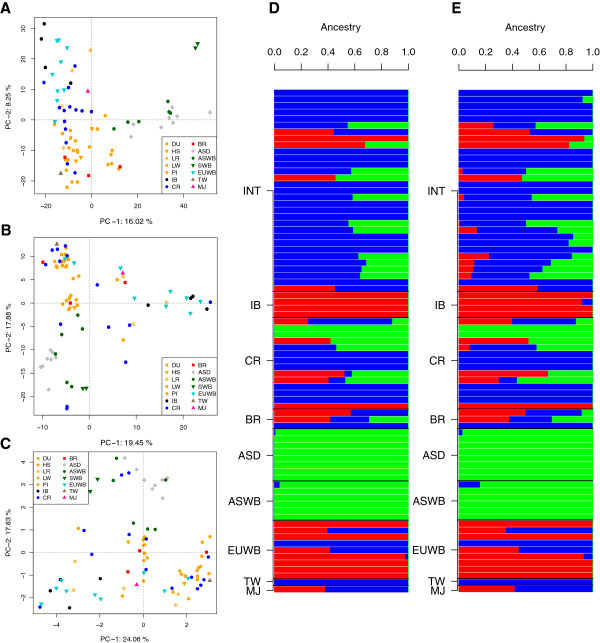
**Principal components (PCA) and structure analysis.** PCA performed on all SNP data from all 19 genes considered **(A)** and for a subset different subsets: bitter **(B)** and non-synonymous SNPs for bitter **(C)**. Structure analyses with full set of SNPs **(D)** and non-synonymous SNPs from bitter taste receptors **(E)**. Each individual is represented by one vertical line with the proportion of assignment to each cluster shown on the top and the different colours are referent to different clusters: international (INT) = blue; Asian (ASD and ASWB) = green; and European (IB and EUWB) = red. Breed and population codes are as in Table 1.

We used Principal Component Analysis (PCA) and STRUCTURE [22] to represent breed and geographic differentiation (Figure 3). We applied PCA at three resolution levels: using all SNPs, using only bitter receptor SNPs, and using only non-synonymous mutations in bitter receptor genes. Both the PCA and STRUCTURE analyses (Figure 3) show a separation between Asian and local European breeds (Iberian) as is typically observed in mtDNA and at the autosomal level [23, 24]. Remarkably, we observed a continuum rather than a clear geographic split between Asia and Europe. For instance, Figure 3A shows the PCA plot with all SNPs from the 19 genes. Here, the first axis explains 16.02% of the variance, and discriminates between Asia and Europe with international breeds clustering in between, but there is a continuum rather than an abrupt divide (e.g. the reader is invited to contrast our Figure 3A with Figure 1 in [25]). The second axis in Figure 3A accounts for 8.25% variance and separates highly selected breeds from wild boar and non-selected local breeds (Iberian and Chinese, the latter to a lesser extent). This suggests that modern selection has exerted a consistent influence across breeds on the pattern of variability in the pig. Creole and Brazilian pigs tend to fall within the international cluster (Figure 3A). This pattern is exacerbated when only bitter receptor polymorphisms are considered (Figure 3B), with some interesting changes: the first axis (19.45% of the variance) now separates European Wild Boar and Iberian from the rest, while the second axis (17.88% variance) distinguishes Asia from the rest. Some international breeds such as Large White are tightly clustered due to their low nucleotide diversity that was smaller than average for this gene groups (π_t_ =1.9 × 10^-3^ ± 1.0 × 10^-3^), may be as a result of a selective pressure in commercial breeds on *Tasrs*. Similar results were observed when only non-synonymous SNPs are employed (Figure 3C).

The STRUCTURE analyses of SNP data from bitter taste receptors suggested that the optimal values of genetic clusters K were 3 for non-synonymous SNP and 3 to 4 for the full set of SNP (Additional file 2). For K =3, Figure 3D and E show a clear separation between Asian (ASD and ASWB), European (IB and EUWB) and international (INT) breeds. Nonetheless, there was a large heterogeneity among individuals within each breed, as is also evident from the PCA graphs. In the Brazilian population (BR), the Piau breed was assigned to the International cluster with 100% probability, whereas Monteiro and Moura present an admixed fraction of genome from Asian and European origins. Within EUWB, three individuals from the Netherlands and France (WB21M03, WB22F02 and WB25U11) were predicted to hold a high international breed component value (>65%). This could be due either to introgression of international breeds into wild boar or to a lack of differentiation between EUWB and international pigs for these genes. In principle, the introgression hypothesis seems a plausible one, given that admixture events between EUWB and domestics have been repeatedly documented [26, 27].

For both set of SNPs the Iberian population was assigned to a cluster of its own, and only one individual had approximately 3% of its genome composition assigned to the International (INT) cluster. This means that the Iberian population is highly homogeneous, presumably because the individuals studied belong to a highly inbred herd.

## Discussion

Our data defines for the first time the full GPCR nutrient and taste receptor gene repertoire in the pig using human and mouse gene sequence homology analysis and a comprehensive survey of its worldwide variability using shotgun sequence data from 79 pigs. However, it should be noted that current porcine assembly 10.2 and its annotation are still incomplete, where about 8% of genome is estimated to be missing [2]; further there is a high missing rate in the NGS data as well (Table 2). In addition, novel nutrient sensing genes might be identified in the future. Thus, future studies may be able to uncover a potential hidden fraction of TR and additional TR variability. Within the *Tasr* repertoire, two main categories have been outlined: those receptors that sense nutrients (the *Tas1r*s, the amino acid-related receptor genes and the fatty acid receptor genes), also referred here as non-bitter TRs (or non-bitter *Tasrs*); and those receptors that sense primarily non-nutritional or potentially toxic compounds known as bitter taste receptors (*Tas2rs*). Admittedly, there is a wide range of non-toxic potential bitter TR ligands including amino acids, peptides or polyphenols amongst many others, but a more detailed discussion on that is outside the scope of the current paper.

Our results from porcine tongue mRNA abundance confirm that the large majority of the genes studied are expressed. The samples were collected by specifically targeting the taste papilla, however, small portions of surrounding structures and cell types (i.e. epithelial cells and underlying muscle tissue), may have also been harvested. Consequently, it is possible (yet unlikely) that the results of the gene transcripts are not related to taste sensory cells. The relative gene expression levels were found to differ significantly (P <0.05) amongst genes. *Tas1r3, Tas2r134 and GPR92* showed the highest whilst *Tas2r1* and *CaSR* were amongst the lowest expression levels. Within the *Tas1rs*, the high relative expression level of *Tas1r3* compared to the other 2 genes supports previous findings that this gene encodes one part of a dimer for both sweet (*Tas2r1* + *Tas1r3*) and umami (*Tas1r2* + *Tas1r3*) taste receptors [28]. The heterodimeric porcine umami receptor (T1r1/T1r3) was the first porcine TR to be sequenced, cloned and fully characterized [29–31]. In agreement with previous reports our data supports the view that pig *Tas1rs* and *mGluR1* have a high homology with the human orthologs [30, 32]. Furthermore, the homology of the porcine *Tas2r3* to the human *TAS2R3* and to the mouse *Tas2r137* has also previously been reported [33].

Other published work on porcine *Tasr* expression have been related to the presence of the receptor proteins T1r2 and T1r3 in the small intestine [34], the presence of the amino acid/peptone receptors GPRC6A, GPR92 and CaSR in gastric antrum [35] and seven *Tas2rs* found to be expressed in five sites of the gastrointestinal tract [36]. However, to our knowledge, these is the first systematic study on porcine *Tasr* expression related to the oral cavity (circumvallate papillae in the tongue) which includes an updated pig taste and nutrient GPCR receptor repertoire. We completed a detailed investigation of the annotation of all the *Tasrs* resulting in the identification of several incorrect intron-exon boundaries and open reading frames in the current porcine annotation for 11 genes. A correct GFF file with the correct annotation is provided in as Additional file 4. All non-bitter *Tasrs* found in the pig genome were annotated as complete functional genes, with the exception of *GPR84*. This gene has a discrepancy in its annotation as NCBI denotes it to be a validated protein coding gene, whereas Ensembl classifies it as a pseudo gene. Our data showing relatively high expression level of this gene seems to further support the NCBI annotation. In contrast, four of the porcine *Tas2rs* were annotated as pseudo genes.

Mammalian diversity in *Tasrs* has been related to dietary adaptations [8, 37]. Consequently, it is tempting to speculate that the differences in *Tasr* homologies, particularly related to fatty acid and amino acid sensing, between humans and pigs might be related to diet. The amino acid and peptone receptors showed the highest homologies between pigs and humans (and also mice) which presumably highlights the nutritional relevance of dietary protein across species. Dietary energy is the other macronutrient essential for life and is mainly related to fats and sugars. In our study, the *Tasrs* for simple carbohydrates (*Tas1r2* and *Tas1r3*) and fatty acids (*GPR40*, *GPR43*, *GPR84* and *GPR120*) except *GPR41* also resulted in high homologies between the pig, humans and mice. Both humans and pigs are omnivorous species. However, pigs in the wild are foraging animals with a diet consisting roughly 90% of plant-derived foods primarily fruits, roots, leaves and grasses. The relative amount of dietary protein and fats from animal tissues is usually well below 10% [38] which accounts for an important difference relative to humans who have evolved on dietary habits containing 30 to 80% of animal-derived foods [39]. A higher reliance on plant-derived foods might, in turn, be related to a higher olfactory acuity of pigs compared to humans and other mammals. To date, pigs have the largest of the olfactory gene repertoire of all studied mammalian species. It might be speculated that the lower number of *Tas2rs* in pigs compared to humans is related to a higher dependency on olfaction. However, the porcine non-bitter (nutrient sensing) *Tasr* repertoire is very similar in number of genes and sequence homology to the human system. In addition, the lower number of porcine *Tas2rs* compared to humans, may not imply a decreased sensitivity to dietary bitter compounds since some of the additional human T2R seem to be narrowly tuned and may not even be related to food volatiles [40]. Overall it seems that pigs have a similar gustatory capacity when compared to humans.

Our results show that the highest degree of divergence between pigs and humans is related to the *Tas2r* repertoire. Humans have 25 functional *TAS2Rs* while our study shows that pigs have only 15 of which 4 have been annotated as pseudo genes (Additional file 1). Of the 25 known human *TAS2Rs*, *TAS2R5* had no porcine gene ortholog and seven of the human *TAS2Rs* had high homology with the porcine *Tas2r20*. The *Tas2r20* is currently annotated in NCBI as a pseudo gene; however, we have several reasons in support of this gene being protein coding. We observed that the porcine *Tas2r20* shares high homologies of up to 77% with the human orthologs (Additional file 1). In addition, when translating the mRNA sequence, we predict 311 amino acids as well as 7 conserved transmembrane domains, both attributes consistent with all the other porcine *Tas2rs*. Furthermore, we have found the gene expression level for *Tas2r20* in pig tongue to be similar to other pig *Tas2rs*. Finally, looking across mammalian species, *Tas2r20* is annotated as a protein coding gene in humans (*Homo sapiens*), chimpanzee (*Pan troglodytes*), mouse (*Mus musculus*), hedgehog (*Echinops telfairi*) and shrew (*Sorex araneus*), to name but a few. We cannot conclusively rule out the possibility that the currently annotated *Tas2r20* is in fact a pseudo gene which would indicate that the *Tas2R20* we have found expressed is currently not annotated.

In contrast, we could not find a human ortholog for porcine *Tas2r1* or *Tas2r134*. Our results show also significant divergence between pigs and mouse such that there was no mouse ortholog for porcine *GPR41*. In addition, of the 35 known mouse *Tas2rs*, 6 had no porcine orthologs. Our findings outlining the bitter taste receptor repertoire in the pig are consistent with a previous report by Groenen et al. [2], with the exception of 3 genes; since we have excluded *Tas2r7A* and *Tas2r7B* and *Tas2r40*. The Gene ID entries in NCBI for *Tas2r7A* and *Tas2r7B* have been discontinued and *Tas2r7C* has been re-annotated as *Tas2R7*. On the other hand, there is an inconsistency in the annotation of the porcine *Tas2r40* between NCBI and Ensembl. In NCBI, the gene appears shorter than the rest of the protein coding porcine *Tas2rs* while Ensembl denotes 3 exons, a feature not related to *Tas2rs*. In addition, *Tas2r40* was annotated in a contig and not in any of the porcine chromosomes. However, differences in the *Tas2r40* sequences between commercial and local pig breeds have been recently reported which should warrant further research [41].

T2Rs are involved in detecting potential toxic compounds, consequently a high plasticity at the gene sequence level suggests a role in the adaptation to different ecosystems and feeding regimes [33, 42]. Different T2Rs respond to different types of bitter tastants and with different ranges [40]. Therefore it is reasonable to envisage that changes in the types and amounts of bitter compounds encountered in a specific environment may elicit specific selection pressures on *Tas2rs*. Recent evidence has shown a dynamic eco-evolutionary process between the bitter taste system and dietary diversity across vertebrates [8], particularly mammalian species [37]. Li and Zhang 2013 [8] showed that the number of genes of the bitter taste system is species dependent and correlates with the relative amount of plant-derived foods usually present in their diet since most potentially toxic compounds are found in plant tissues. Consequently, it might be inferred that dietary toxins play an important selection driver shaping between-species *Tas2r* diversity. Our pig population genomic analysis showed that *Tas2rs* exhibited higher nucleotide diversity than both fatty acid and amino acid receptors (Tables 3 and 4). In addition, this gene variability is higher than the normally reported for the pig species genome wide. These findings provide additional evidence of the potential role of the bitter taste system in the adaptation, possibly through balancing selection, to various ecological niches in agreement with recent findings related to mammalian species [37].

In general, the high average nucleotide diversity in gene regions compared to intergenic regions for *Tas2rs* was in contrast to the remaining groups of genes, which showed the opposite effect. The incidence and location of the non-synonymous SNPs across the 10 porcine *Tas2rs* occur with the same frequency in both the transmembrane and non-transmembrane domains indicating that there has been no selection signature for having mutations in predicted ligand binding domains. Among non-synonymous variants, the most potentially deleterious ones, according to the SIFT score, were in general at low frequency (Additional file 2). An interesting exception was that of nsSNP rs330666697 (*Tas1r1*), with intermediate frequency in Asian domestics and segregating in international breeds as well. The high frequency in Asian domestics but absence in Asian wild boars suggest that this mutation appeared after domestication and that quickly raised in frequency afterwards, may be because its potentially deleterious consequences were offset by other advantages and was positively selected. Further functional studies are required to confirm this hypothesis. Using homology analysis with the *TAS1R1* human sequence, the pig SNP rs330666697 is predicted to be located in the first transmembrane helical domain. The polymorphism is unlikely to affect ligand binding because the ligand binding domain in *Tas1r1* is located in the extracellular N-terminus [28, 43]. Furthermore, the amino acid change L- > V is unlikely to have significant consequences (e.g. protein folding) as both AA are non-polar, i.e. hydrophobic.

The Asian and European wild boars diverged *ca*. 1.2 M [2]. This long evolutionary distance results in two highly differentiated clusters when both Asian and European pigs are investigated using, e.g., high density SNP arrays or mitochondrial phylogeny [2, 25]. It is therefore noteworthy that *Tasr* phylogeography departs significantly from the genome wide autosomal pattern and, for these taste receptors, the extreme autosomal Asia – Europe divergence is highly attenuated (Figure 3A). A potential explanation for lack of divergence between Europe and Asia would be the well-known introgression of Chinese pigs into European domestics that occurred as of the 17th century onwards, followed by selection of Chinese haplotypes. Although this has been observed in some genes [44], it is unlikely to be the (main) reason for the pattern observed since a high variability is found across all populations, including European wild boar. Nevertheless, Asian introgression cannot be excluded. To study this issue better, we carried out a PCA and computed neighbor-joining (NJ) trees for each individual gene (results not shown). Interestingly, for the most differentiated gene, Tas2R9 (F_ST_ = 0.41), the NJ tree (Additional file 5) does suggest the presence of introgression in Large White and Hampshire, as well as in some Creole pigs,

Assuming that the genome wide pattern is primarily the result of drift, a less than expected differentiation might be explained by some sort of balancing selection at the TR genes. Balancing selection could also explain that variability is higher than genome wide and that remains approximately constant within the *Tasrs* and the flanking regions (Table 3). A higher than expected variability could be an artifact due to the presence of copy number variants (CNVs) However, this is unlikely in this case since we did not find any overlap between *Tasr* positions and CNV coordinates reported in the pig genome [20, 45, 46]. In contrast, purifying selection seems also to have played a role in shaping *Tasr* diversity, given the prevalence of ratios of non-synonymous to synonymous nucleotide diversity (π_a_/π_s_) smaller than one (Table 3). These results do not seem to agree, in part, with Groenen et al. [2], who found four taste receptor genes (*Tas1r2*, *Tas2r1*, *Tas2r40* and *Tas2r39*) under positive selection (π_s_/π_a_ ratio equal to 1.5 to 1.9). However fewer samples were used in the previous study compared to the current data set, which includes Creole, Brazilian and local Iberian pigs. To verify this result, we also computed other tests for detection of positive selection (i.e. the HKA [47] and the McDonald-Kreitman [48] tests), but none of them were significant (results not presented), suggesting weak or no positive selection pressure.

Genome wide analyses have shown a higher nucleotide diversity in Asia than in Europe, as expected due to the bottleneck experienced by European wild boars when migrating out of Asia [49]. In Asia, a reduced diversity in domestics vs. wild boars was also observed by Bosse et al. [49] and Groenen et al. [2]. Interestingly, this reduction in diversity was not observed for taste receptors neither when comparing Asian vs. European wild boars, nor between Asian domestics and wild boars (Table 4, Additional file 3). The only population with a marked reduction in diversity was the Iberian breed, and it should be mentioned that the strain sequenced here pertains to a closed population (Guadyerbas) maintained genetically isolated since 1945 [21]. As argued by Esteve-Codina et al. [20], inbreeding due to confinement explains most of loss in variability in this strain, whereas the whole of Iberian strains hold a variability comparable to that found today in European wild boar. The most variable populations were American village pigs (Creole and Brazilian); this apparently surprising finding can be explained by their admixed nature, as these pigs are the result of crossing with many different origins [25].

## Conclusions

We are defining a full GPCR-based nutrient and taste receptor gene repertoire in the pig and a comprehensive analysis of its worldwide variability using shotgun sequence data from 79 domestic and wild pigs of 14 different breeds. The porcine *Tasr* repertoire in our study consists of 28 genes of which 15 have been identified as bitter taste receptor genes (*Tas2rs*) of which 4 were pseudo genes. Our findings on *Tasrs* improve the most recent annotation of the pig genome (*Sus scrofa 10.2*). In addition, all the researched genes (except *Tas2r40*, for reasons discussed) were found to be expressed at different levels in pig’s tongue circumvallate papillae. Our pig population genomic analysis showed that bitter taste genes had higher nucleotide diversity than either fatty acid or amino acid receptors. The cluster of genes related to bitter taste (*Tas2rs*) showed the lowest degree of homology with the human repertoire together with the highest nucleotide diversity when compared to the fatty acid and amino acid receptors. These findings are interpreted as evidence of a dynamic eco-evolutionary process between the bitter taste system and dietary adaptation particularly to plant compounds. Interestingly, we also found a much less marked divergence between Asian and European haplotypes than found with genome wide markers; that, together with the high variability, may be indicative of a balancing selection at these loci, in particular for bitter taste receptors.

## Methods

### Ortholog identification and verification

The mRNA sequences of all 25 and 36 known bitter taste receptors for human and mouse respectively were obtained from NCBI. In addition, mRNA sequences for known fatty and amino acid receptors were also collected from human and mouse databases. Each one of these sequences was blasted to the pig refseq genome assembly using the megablast algorithm. Only when no hits were found, was a less conservative method used in a step-wise fashion from discontiguous megablast to the blastn algorithm. Genes were considered orthologous according to the criteria that the identity percentage was equal to greater than 50%. In addition, specific to *Tas2rs*, only genes with a single exon of approximately 300 amino acids in length were considered for the gene expression experiment and SNP analysis.

In order to verify correct annotation of open reading frames of the identified porcine candidate taste receptor genes, the mRNA sequences were downloaded from NCBI and checked as follows. The nucleotides were translated into amino acids using the online software ExPASy (http://www.expasy.org/) [50]. Using this information start and stop sites were up-dated where appropriate and can be seen in Additional file 6.

### Gene expression analysis

The real time PCR assays were carried out according to previously defined requirements [51]. PCR primers were designed in order to specifically amplify unique fragments of each of the pig taste receptor genes that were identified in the BLAST analysis. We acknowledge that the primers for *GPR92* were previously published [35]. In addition, primers for the two reference genes, *RPLP* and *β-actin* were also designed. The details of these primers are included in Additional file 6. The specificity of the primers was established by confirming single products of the correct gene was amplified by a PCR blast in NCBI, as well as by the presence of single bands of the correct size of PCR products run on agarose gels. Furthermore, the melt curves from the real time PCR reactions were singular and sharp, indicating single products, with no evidence of secondary structures that could inhibit the PCR. The relative gene expression levels were estimated using the Pfaffl method [52] which involved taking into account the cycle threshold (CT) values of both the candidate genes and of the two reference genes, as well as taking into account the efficiency of each of the primer sets. These normalized values were then standardized to a calibrator assay, *Tas1r1* expression in the circumvallate papillae. In order to identify which of the receptor genes identified in the pig taste repertoire are expressed, tongue tissues were collected from 6 newly weaned piglets (24 ± 3 days of age and 9.367 ± 2.7 kg of body weight) following exsanguination (animal ethics approval: CNFS/217/11/PORK CRC). The 6 piglets (3 males) represent biological replicates from the same breed (Large White) and were equally reared following standard pig production practices at the University of Queensland, Gatton piggery. From these tongues, circumvallate papillae were isolated and total RNA was extracted using a TRIZOL-chloroform method, where RNA is purified using a Qiagen RNeasy column, followed by a sodium acetate cleanup step. The RNA was reverse transcribed into cDNA using a Qiagen Reverse transcription kit. No-reverse transcription controls were included to ensure that there was no genomic contamination present. The real time qPCR assays were carried out using SYBR green in a ViiA™ 7 Real-Time PCR system (Applied Biosystems, Life Technologies).

### Sampling and sequencing

Whole genome shotgun sequences of 77 pigs from international, American Creole, European and Asian domestic breeds, Asian and European wild boars was analyzed in this study. We also included two Sumatran wild boars as out-groups. Of those sequences, 54 were downloaded from SRA accession numbers [20, 27, 53, 54] and 25 are unpublished. New sequences were obtained with HiSeq Illumina’s technology, paired end reads of 100 base pairs (bp) long. The new genomes, primarily Iberian pigs and American village (Creole) pigs, were a subset of those described previously [25]. Samples were grouped into international (comprising the well-known highly selected breeds Large White, Landrace, Duroc, Pietrain and Hampshire), Creole (village) pigs from several American countries, local breeds from Brazil (Moura, Monteiro and Piau), Chinese breeds (Meishan, Xiang, Jiangquhai and Wuzhishan) and Wild Boars from Europe and Asia (Table 2). We directly downloaded the aligned bam files for the samples in [53]; for the remaining sequences, we aligned the reads using Burrows Wheeler Alignment tool (BWA) [55] allowing for 7 mismatches per 100 bp long read.

### SNP calling and annotation

Pig candidate genes analyzed in this study included all 21 available taste receptors in assembly *Sus scrofa* genome built 10.2 for bitter (*Tas2r7*, *Tas2r9*, *Tas2r10*, *Tas2r16*, *Tas2r20*, *Tas2r38*, *Tas2r39*, *Tas2r40*, *Tas2r41*, *Tas2r42* and *Tas2r60*), amino acid receptor (*GPRC6A*, *mGluR1*, *mGluR4*, *Tas1r3*, *Tas1r1* and *CaSR*) and fatty acid receptors (*GPR40*, *GPR43*, *GPR41*, *GPR120* and *GPR84*). Six genes were excluded from the analysis (*Tas1r2*, *Tas2r1*, *Tas2r134*, *Tas2r3*, *Tas2r4 and GPR92*), because they were not present in the official assembly or were in isolated scaffolds.

First, single nucleotide polymorphisms (SNPs) were called for each sample individually using SAMtools v.0.0.18 mpileup function [55], filtering by base and mapping qualities of at least 20. Minimum and maximum depths were set to five and twice the average depth per sample, respectively. The Variant Call Format files version 4.0 (VCF) resulting from the SNPs calling were then merged into a multi individual VCF using custom Perl scripts. For missing positions, the bam files were inspected to check whether the reference allele was present (always filtering by the same quality criteria as above) and the VCF file was completed if possible. Otherwise the position was treated as missing. After obtaining the joint VCF file, the region of interest of the 21 candidate genes distributed among pig chromosomes 1, 5, 6, 7, 13, 14 and 18 were obtained to analysis from these smallest windows, and 10 kb flanking regions according to reference gene coordinates (*Sus scrofa* 10.2) were added. If two genes were closer than 20 kb, the intergenic region was split in half and ‘assigned’ to each corresponding gene.

Each SNP was annotated with Variant Effect Predictor (VEP) perl script tool available in Ensembl http://www.ensembl.org/info/docs/tools/vep/index.html [56], using Ensembl database v. 72. This was done only for those genes (*Tas2r9*, *Tas2r39*, *Tas2r41*, *Tas2r60*, *GPRC6A*, *mGluR1*, *mGluR4*, *Tas1r1*, *Tas2r3*, *CaSR*, *GPR40*, *GPR43*, *GPR41*, *GPR120* and *GPR84*) where the official annotation coincided with our manually obtained annotation. Standard settings including the options Sorting Intolerant From Tolerant (SIFT), to predict the effect of amino acid substitution on protein function [57] for non-synonymous SNPs (nsSNPs), and to check for existent co-located variants that returns the reference SNP ID number (rsID) from database of SNP (dbSNP) were included. For the remaining genes, SNP class (in intergenic, exonic, intronic, and in untranslated regions (UTRs) as well as consequence of variations in transcripts), was assessed either manually or with mstatspop program v.0.998978b, S. Ramos-Onsins, unpublished, available at http://bioinformatics.cragenomica.es/numgenomics/people/sebas/software/software.html). A customized GFF3 v3 file and FASTA files corresponding to each gene were generated using custom PERL scripts. The FASTA files, where missing positions are replaced by N’s, were used as input for mstatspop program.

### Statistics analysis

We calculated the global and by population allele frequency for each SNP with VCF tools program version 0.1.11 [58] and the mstatpop program was used to estimate percentage of missing data and diversity parameters such as total nucleotide diversity (π_t_), i.e., considering the full region that included genic and intergenic region, genic (π_g_), intergenic region (π_int_), intron (π_i_), exons (π_e_) for genes with more than one exon (for those genes with only one exon the nucleotide diversity is the same found in genic region), and in UTRs regions (π_utr_). The rate of synonymous (π_s_) and non-synonymous (π_a_) variability rates were performed to investigate selection pressure on taste receptor genes (π_a_/π_s_). A ω = π_a_/π_s_ ratio >1 is indicative of a long term pattern of positive selection, whereas less than one suggest purifying selection, and a ratio of one may indicate neutrality [59]. Fixation index (F_ST_ =1-π_iw_/π_it_), where π_iw_ is the average number of different nucleotides between two sequences within populations and π_it_ is the number of different nucleotides in the whole population was obtained. Its significance was computed with 1000 permutations. Approximate standard errors (SE) of nucleotide diversities for each gene were obtained by generation of 95% confidence interval (CI) including 1000 random samples and using by default an intermediate recombination rate model (R =10) using the neutral coalescent simulator in DnaSP v5 [60]. For these simulations, we used estimates of nucleotide variability, diversity and number of sites corrected for missing computed with mstatspop.

Principal component analysis (PCA) was conducted in R software [61] (http://cran.rproject.org) with PLINK format files [62] extracted from VCF file using a custom Perl script. This analysis was performed on the full SNPs set and on the non-synonymous sites set as well as on the different genes groups (bitter, amino acid and fatty acid taste receptors) to study genetic structure of the population. We also investigated the genetic relationships with STRUCTURE version 2.3.4 [22]. We performed a structure analysis with two sets of SNPs in bitter taste receptor genes: (1) including only nsSNP and (2) a set composed of SNPs from noncoding and coding region. We performed five permutations for each number of populations (K) that ranged from 1 to 15 with 100,000 MCMC (Markov chain Monte Carlo) and a burning period of 10,000 steps and employed admixture and correlated allele frequency parameters. The significant *K* number of different genetic clusters was obtained by the Delta K statistic [63] which was calculated using STRUCTURE HARVESTER version 0.9.93 [64]. Genetic distances were calculated with PLINK software using the SNPs data from each gene, and then we used this information to create Neighbor-Joining trees using R. The trees will help us to visualize the genetic differences between the individuals from different locations and breeds in the world, as well as if there was indication of Asian haplotypes into international pig breeds as a result of the introgression process.

### Ethics Statement

Animal care and procedures were performed following the Australian Animal Welfare Standards and Guidelines (http://www.daff.gov.au/animal-plant-health/welfare/standards-guidelines) [65] and approved by the Animal Ethics Committee of the University of Queensland (Approval Certificate: CNFS/217/11/PORK CRC).

### Availability of supporting data

New SNPs identified have been submitted to dbSNP (accession application in progress).

## Electronic supplementary material

Additional file 1: **The 28 taste receptor genes identified for the pig.** Shown is the gene annotation information from NCBI, as well as a summary of the BLAST results for the human and mouse genomes. (DOCX 26 KB)

Additional file 2: **Complete list of SNPs, with rs id if in dbSNP gene, position, alternative allele, SIFT prediction for non-synonymous changes, and allele frequency by population.** SIFT score ≤0.05 is considered as potentially deleterious in the protein function and values >0.05 are tolerated. (TXT 1 MB)

Additional file 3: **Nucleotide diversity for total (π**_**t**_ **× 10** ^**3**^ **) and genic region (π**_**g**_ **× 10** ^**3**^ **) by population.** (DOC 73 KB)

Additional file 4: **The General Feature Format (GFF) file used as input for analysis of SNPs.** *denotes updates compared to current annotation. (TXT 13 KB)

Additional file 5: **NJ tree of genetic distances for Tas2R9 gene.** Color triangles represent population origins: INT, International; IB, Iberian; CR, Creole; BR, Brazilian; ASD, Asian domestic; ASWB, Asian wild boar; EUWB, European wild boar; SWB, Sumatran wild boar. The first two letters of each sample are the breed code: CR, creole; LR, Landrace; LW, Large White; IB, Iberian; HA, Hampshire; XI, Xian; MS, Meishan; JQ, Jianquahi; TW, Tamworth; DU, Duroc. Note, eg, that six out of 14 LW samples cluster near Asian samples, together with some Creole and Pietrain individuals. (PDF 231 KB)

Additional file 6: **Primer details for the porcine nutrient sensing and taste receptor genes used for estimating relative gene expression levels.** (DOCX 19 KB)

## Abbreviations

ACTB: Beta-actin gene
ASD: Asian domestic population
ASWB: Asian wild boar population
ca: Circa (approximately)
CI: Confidence interval
CNV: Copy number variant
CR: Creole population
EUWB: European wild boar population
GPCR: G-Protein coupled receptor
IB: Iberian population
INT: International population
mtDNA: Mitochondrial DNA
MYA: Million years ago
NJ: Neighbor-joining
NGS: Next generation sequence
Ns: Non-synonymous
nsSNP: Non-synonymous single nucleotide polymorphism
PCA: Principal component analysis
PUFA: Polyunsaturated fatty acid
qPCR: Quantitative PCR
RPLP: Ribosomal subunit protein gene
SIFT: Sorting Intolerant from tolerant
Sy: Synonymous
TR: Taste receptor gene
Tasr: Nutrient sensing and taste receptor gene
Tas1rs: Family 1 taste receptor genes
Tas2rs: Family 2 taste receptor genes
ti/tv: Average rate of transitions vs transversions
T1R: Family 1 taste receptor
T2R: Family 2 taste receptor
UTR: Untranslated region
VCF: Variant call format
VEP: Variant effect predictor
WB: Wild boar population
Πs: Synonymous variability rate
Πa: Non-synonymous variability rate.
