# Supplementary material for: Characterization of the porcine nutrient and taste receptor gene repertoire in domestic and wild populations across the globe
Source: BMC Genomics. 2014 Dec 3;15(1):1057. doi: 10.1186/1471-2164-15-1057 (PMC4302110; doi:10.1186/1471-2164-15-1057)
Supplement: Supplementary file 1 — Additional file 1: The 28 taste receptor genes identified for the pig. Shown is the gene annotation information from NCBI, as well as a summary of the BLAST results for the human and mouse genomes. (DOCX 26 KB) [file 12864_2014_6798_MOESM1_ESM.docx]

**Additional file 1.** **The porcine taste and nutrient receptor genes with the main human and mouse orthologs**. Shown is the gene annotation information from NCBI, as well as a summary of the BLAST results for the human and mouse genomes.

| *S. scrofa* gene^#^ | Gene type (*S. scrofa*) | Refseq status (*S. scrofa)* | Human | | Mouse | |
| --- | --- | --- | --- | --- | --- | --- |
|  |  |  | Orthologous gene | Homology % | Orthologous gene | Homology % |
| *Tas1r1* | protein coding | MODEL | *TAS1R1 (variant 2)^a^* | 84% | *Tas1r1^b^* | 79% |
| *Tas1r2* | protein coding | PROVISIONAL | *TAS1R2* | 84% | *Tas1r2* | 79% |
| *Tas1r3* | protein coding | PROVISIONAL | *TAS1R3* | 82% | *Tas1r3* | 76% |
| *Tas2r1* | pseudo gene | MODEL | *No gene* | n/a | *No gene* | n/a |
| *Tas2r3* | protein coding | MODEL | *TAS2R3* | 82% | *^*^Tas2r137* | 77% |
| *Tas2r4* | protein coding | MODEL | *^*^TAS2R4* | 80% | *^*^Tas2r108* | 74% |
| *Tas2r7^#^* | protein coding | MODEL | *TAS2R7* | 83% | *Tas2r130* | 81% |
| *Tas2r9* | protein coding | MODEL | *TAS2R9* | 82% | *^*^Tas2r130* | 65% |
| *Tas2r10* | protein coding | MODEL | *TAS2R10^c^* | 84% | *Tas2r106^d^* | 75% |
| *Tas2r16* | protein coding | MODEL | *^*^TAS2R16* | 76% | *^*^Tas2r118* | 71% |
| *Tas2r20* | pseudo gene | MODEL | *TAS2R19^e^* | 77% | *^*^Tas2r120^f^* | 69% |
| *Tas2r38* | protein coding | MODEL | *^*^TAS2R38* | 78% | *^*^Tas2r138* | 75% |
| *Tas2r39* | pseudo gene | MODEL | *TAS2R39* | 82% | *^*^Tas2r139^g^* | 71% |
| *Tas2r40* | pseudo gene | MODEL | *TAS2R40* | 78% | *^*^Tas2r144* | 73% |
| *Tas2r41* | protein coding | MODEL | *^*^TAS2R41* | 79% | *^*^Tas2r126* | 77% |
| *Tas2r42* | protein coding | MODEL | *^*^TAS2R42* | 76% | *^*^TAS2r42-like^h^* | 64% |
| *Tas2r60* | protein coding | MODEL | *^*^TAS2R60* | 77% | *^*^Tas2r135* | 72% |
| *Tas2r134* | protein coding | MODEL | *No gene* | n/a | *^*^Tas2r134* | 73% |
| *GPR120* | protein coding | PROVISIONAL | *GPR120 (variant 2)^i^* | 88% | *GPR120* | 84% |
| *GPR40* | protein coding | PROVISIONAL | *GPR40* | 84% | *^*^GPR40* | 81% |
| *GPR43* | protein coding | PROVISIONAL | *GPR43* | 86% | *GPR43 (variant 1)^j^* | 81% |
| *GPR41* | protein coding | MODEL | *^*^GPR41* | 75% | *No gene* | n/a |
| *GPR84* | protein coding | VALIDATED | *GPR84* | 88% | *GPR84^k^* | 84% |
| *mGluR1* | protein coding | MODEL | *mGluR1 (variant X1)^l^* | 92% | *mGluR1 (variant 1)^m^* | 89% |
| *mGluR4* | protein coding | MODEL | *mGluR4 (variant X2)^n^* | 93% | *mGluR4 (variant 2)^o^* | 91% |
| *GPRC6A* | protein coding | MODEL | *GPRC6A (variant 2)^p^* | 90% | *GPRC6A^q^* | 84% |
| *CaSR* | protein coding | INFERRED | *CaSR (variant 1)^r^* | 91% | *CaSR^s^* | 88% |
| *GPR92* | protein coding | MODEL | *GPR92 (variant 2)^t^* | 84% | *^**^GPR92* | 78% |

Genes with no asterisk were obtained from using the BLAST algorithm “Highly similar sequences (megablast)”

^*^Genes with one asterisk were obtained from using the BLAST algorithm “More dissimilar sequences (discontiguous megablast)”

^**^Genes with two asterisks were obtained from using the BLAST algorithm “Somewhat similar sequences (blastn)”

*^a-t^* Alphabetic letter superscripts indicate additional ortholog(s) to the main gene in the table and are listed in alphabetical order with the % homology in brackets. Human TR gene orthologs are written with full capital letters (e.g. *TAS1R* or *TAS2R*) while mouse gene orthologs are written with lower case gene symbol (e.g. *Tas1r* or *Tas2r*):  *^a^TAS1R1 variant 3* (81%); *^b^Tas1r1* *variant X1* (79%), *Tas1r1 variant X2* (79%); *^c^TAS2R10 variant X1* (84%); *^d^Tas2r114* (71%), *Tas2r104* (73%), *Tas2r105* (73%), *Tas2r107* (73%) ,; *^e^*TAS2R14 (74%),  *TAS2R20* (76%), *TAS2R43* (76%), *TAS2R45* (76%), *TAS2R46* (76%), *TAS2R31* (77%), ; *^f^Tas2r117* (65%), *Tas2r123* (68%),; *^g^Tas2r144* (71%); *^h^Tas2r131* (64%); *^i^FFAR4/GPR120 variant 1* (87%); *^j^GPR43 variant 2* (81%), *GPR43 variant 3* (81%), *GPR43 variant 4* (81%), *GPR43 variant 5* (81%); *^k^GPR84 variant X1* (84%); *^l^mGluR1 variant X2* (92%), *mGluR1 variant 3* (92%), *mGluR1 variant 4* (92%), *mGluR1 variant 5* (92%), *mGluR1 variant 6* (92%); *^m^mGluR1 variant X1* (89%), *mGluR1 variant 2* (89%), *mGluR1 variant X2* (89%), *mGluR1 variant X3* (89%), *mGluR1 variant X4* (89%), *mGluR1 variant X5* (89%); *^n^mGluR4 variant X1* (90%), *mGluR4 variant 2* (91%), *mGluR4 variant 1* (91%), *mGluR4 variant 4* (91%), *mGluR4 variant 5* (91%), *mGluR4 variant 6* (91%), *mGluR4 variant 8* (91%); *^o^mGluR4 variant 1* (90%), *mGluR4 variant X1* (90%), *mGluR4 variant X2* (90%), *mGluR4 variant X3* (90%); *^p^GPRC6A variant 1* (89%), *GPRC6A variant 3* (89%); *^q^GPRC6A variant X1* (84%); *^r^CasR variant 2* (91%), *CasR variant X1* (91%), , *CasR variant X2* (91%), *CasR variant X3* (91%), *CasR variant X4* (91%); *^s^CaSR variant X1* (88%), *CaSR variant X2* (88%), *CaSR variant X3* (88%), *CaSR variant X4* (88%), *CaSR variant X5* (88%), *CaSR variant X6* (88%), *CaSR variant X7* (86%), *CaSR variant X8* (86%); ^t^*GPR92 variant 1* (83%)

^#^ The current porcine genome annotation contains a second *Tas2r7* classified as a pseudo gene which has been excluded from the table as it had no homology to human or mouse genes.
