# Supplementary material for: Characterization of the porcine nutrient and taste receptor gene repertoire in domestic and wild populations across the globe
Source: BMC Genomics. 2014 Dec 3;15(1):1057. doi: 10.1186/1471-2164-15-1057 (PMC4302110; doi:10.1186/1471-2164-15-1057)
Supplement: Supplementary file 3 — Additional file 3: Nucleotide diversity for total (π t × 10 3 ) and genic region (π g × 10 3 ) by population. (DOC 73 KB) [file 12864_2014_6798_MOESM3_ESM.doc]

| Gene Groups | Genes |  | | International (n=31) | |  | Iberian (n=04) | |  | Creole (n=14) | |  | Brazilian (n=03) | |  | Asian (n=08) | |  | Asian WB (n=06) | |  | European WB (n=09) | |
| --- | --- | --- | --- | --- | --- | --- | --- | --- | --- | --- | --- | --- | --- | --- | --- | --- | --- | --- | --- | --- | --- | --- | --- |
|  |  |  | πt | | πg |  | πt | πg |  | πt | πg |  | πt | πg |  | πt | πg |  | πt | πg |  | πt | πg |
| Bitter | TAS2R20 |  | 1.6±0.6 | | 2.6±0.2 |  | 0.3±0.0 | 0.3±0.0 |  | 2.7±0.3 | 3.8±0.5 |  | 2.4±0.5 | 3.5±0.6 |  | 0.3±0.0 | 0.6±0.0 |  | 2.5±0.7 | 0.7±0.1 |  | 2.7±0.4 | 3.9±1.6 |
|  | TAS2R9 |  | 2.4±0.5 | | 1.1±0.3 |  | 0.7±0.0 | 0.5±0.1 |  | 4.4±1.4 | 1.1±0.0 |  | 2.6±0.6 | 1.7±0.2 |  | 3.7±0.6 | 1.3±0.5 |  | 4.4±0.6 | 1.5±0.4 |  | 1.6±0.3 | 0.6±0.1 |
|  | TAS2R10 |  | 1.9±0.3 | | 0.5±0.0 |  | 1.5±0.5 | 1.7±0.5 |  | 3.3±0.9 | 1.1±0.2 |  | 2.3±0.3 | 1.1±0.1 |  | 2.4±0.2 | 0.1±0.1 |  | 4.6±1.8 | 1.5±0.4 |  | 1.1±0.1 | 1.1±0.1 |
|  | TAS2R42 |  | 1.9±0.3 | | 4.4±0.6 |  | 0.3±0.0 | 1.0±0.2 |  | 3.1±0.6 | 6.3±2.4 |  | 2.5±0.6 | 6.4±2.9 |  | 1.8±0.3 | 4.4±1.9 |  | 2.3±0.3 | 4.2±1.2 |  | 1.9±0.3 | 4.9±0.7 |
|  | TAS2R16 |  | 2.1±0.3 | | 1.5±0.3 |  | 2.2±0.3 | 1.5±0.4 |  | 1.6±0.2 | 1.3±0.1 |  | 7.8±1.8 | 6.0±2.1 |  | 2.2±0.2 | 1.7±0.5 |  | 3.2±0.5 | 1.0±0.1 |  | 6.2±0.7 | 4.4±1.4 |
|  | TAS2R38 |  | 0.8±0.1 | | 5.9±0.7 |  | 0.8±0.1 | 5.6±0.9 |  | 0.9±0.1 | 5.2±0.6 |  | 0.9±0.1 | 3.7±1.0 |  | 0.9±0.0 | 4.6±0.6 |  | 0.7±0.0 | 1.5±0.3 |  | 0.8±0.0 | 6.3±2.6 |
|  | TAS2R39 |  | 2.8±0.5 | | 4.7±1.8 |  | 1.7±0.2 | 3.1±0.8 |  | 3.4±0.5 | 5.4±0.8 |  | 4.1±0.3 | 6.9±1.8 |  | 3.9±0.9 | 5.5±1.9 |  | 3.5±1.1 | 6.5±1.5 |  | 2.0±0.2 | 2.2±0.4 |
|  | TAS2R41 |  | 0.5±0.1 | | 2.4±0.5 |  | 0.6±0.1 | 2.9±0.5 |  | 1.0±0.1 | 3.2±0.8 |  | 0.5±0.1 | 2.0±0.0 |  | 0.8±0.0 | 3.5±0.5 |  | 0.8±0.1 | 4.3±1.6 |  | 0.8±0.0 | 2.6±0.6 |
|  | TAS2R60 |  | 1.0±0.2 | | 3.6±0.3 |  | 1.4±0.3 | 3.3±0.3 |  | 1.5±0.5 | 3.7±1.8 |  | 0.9±0.2 | 2.3±0.3 |  | 1.0±0.0 | 3.0±0.9 |  | 0.6±0.1 | 2.6±0.6 |  | 0.9±0.1 | 2.1±0.3 |
|  | TAS2R7C |  | 3.2±0.3 | | 1.7±0.5 |  | 0.4±0.0 | 0.0±0.0 |  | 4.9±1.3 | 2.4±0.5 |  | 4.0±0.5 | 2.2±0.7 |  | 2.8±0.6 | 1.4±0.3 |  | 2.8±0.8 | 1.5±0.3 |  | 3.2±1.4 | 2.7±0.7 |
| Amino  Acids | mGluR4 |  | 4.9±0.3 | | 5.1±0.5 |  | 0.2±0.0 | 0.2±0.0 |  | 4.2±0.6 | 4.2±0.5 |  | 5.1±0.7 | 5.3±0.9 |  | 3.1±0.9 | 3.0±1.0 |  | 4.4±1.6 | 4.5±0.7 |  | 2.5±0.4 | 2.6±0.3 |
|  | GPRC6A |  | 1.4±0.2 | | 0.2±0.0 |  | 0.2±0.0 | 0.1±0.0 |  | 0.7±0.0 | 0.5±0.0 |  | 1.4±0.3 | 0.9±0.1 |  | 1.9±0.5 | 1.4±0.3 |  | 1.7±0.3 | 1.5±0.3 |  | 0.2±0.0 | 0.1±0.0 |
|  | mGluR1 |  | 2.0±0.5 | | 2.0±0.6 |  | 0.8±0.2 | 0.8±0.0 |  | 1.5±0.6 | 1.5±0.1 |  | 2.5±0.6 | 2.6±0.6 |  | 3.1±1.4 | 3.2±1.2 |  | 3.3±1.2 | 3.3±1.8 |  | 1.7±0.2 | 1.7±0.3 |
|  | Tas1R1 |  | 1.0±0.1 | | 1.1±0.2 |  | 0.4±0.0 | 0.4±0.0 |  | 1.2±0.2 | 1.2±0.3 |  | 1.6±0.2 | 1.7±0.2 |  | 1.2±0.2 | 1.3±0.3 |  | 1.2±0.2 | 1.3±0.2 |  | 0.2±0.0 | 0.2±0.0 |
| Fatty acids | GPR40 |  | 1.3±0.3 | | 0.8±0.1 |  | 0.0±0.0 | 0.0±0.0 |  | 2.9±0.5 | 2.3±0.5 |  | 3.4±0.7 | 2.2±0.7 |  | 1.6±0.5 | 0.9±0.1 |  | 1.6±0.1 | 0.7±0.1 |  | 0.2±0.0 | 0.4±0.1 |
|  | GPR43 |  | 1.2±0.2 | | 0.8±0.1 |  | 0.1±0.0 | 0.0±0.0 |  | 2.6±0.5 | 2.8±0.3 |  | 3.2±0.3 | 2.2±0.3 |  | 2.7±0.5 | 3.3±0.7 |  | 2.6±0.4 | 2.9±0.7 |  | 0.2±0.0 | 0.3±0.0 |
|  | GPR41 |  | 2.5±0.3 | | 0.9±0.2 |  | 0.2±0.0 | 0.0±0.0 |  | 4.3±0.3 | 1.1±0.1 |  | 5.0±0.7 | 1.9±0.2 |  | 2.8±0.4 | 2.8±0.8 |  | 1.3±0.3 | 1.4±0.3 |  | 0.1±0.0 | 0.3±0.0 |
|  | GPR120 |  | 1.6±0.5 | | 2.1±0.5 |  | 1.4±0.2 | 1.2±0.3 |  | 2.1±0.3 | 2.7±0.5 |  | 2.2±0.3 | 2.3±0.5 |  | 2.2±0.3 | 2.7±0.7 |  | 3.0±0.3 | 3.5±0.9 |  | 1.2±0.1 | 1.1±0.3 |
|  | GPR84 |  | 0.4±0.0 | | 0.2±0.0 |  | 0.4±0.0 | 0.0±0.0 |  | 0.6±0.1 | 0.0±0.0 |  | 0.7±0.2 | 0.0±0.0 |  | 1.2±0.4 | 0.0±0.0 |  | 0.9±0.1 | 0.0±0.0 |  | 0.4±0.0 | 0.2±0.0 |
|  | Mean |  | 1.8±0.3 | | 2.2±0.4 |  | 0.7±0.1 | 1.2±0.2 |  | 2.5±0.5 | 2.6±0.5 |  | 2.8±0.5 | 2.9±0.7 |  | 2.1±0.4 | 2.3±0.6 |  | 2.4±0.5 | 2.3±0.6 |  | 1.5±0.2 | 1.9±0.5 |
