# Supplementary figures and images for: Characterization of the porcine nutrient and taste receptor gene repertoire in domestic and wild populations across the globe
Source: BMC Genomics. 2014 Dec 3;15(1):1057. doi: 10.1186/1471-2164-15-1057 (PMC4302110; doi:10.1186/1471-2164-15-1057)

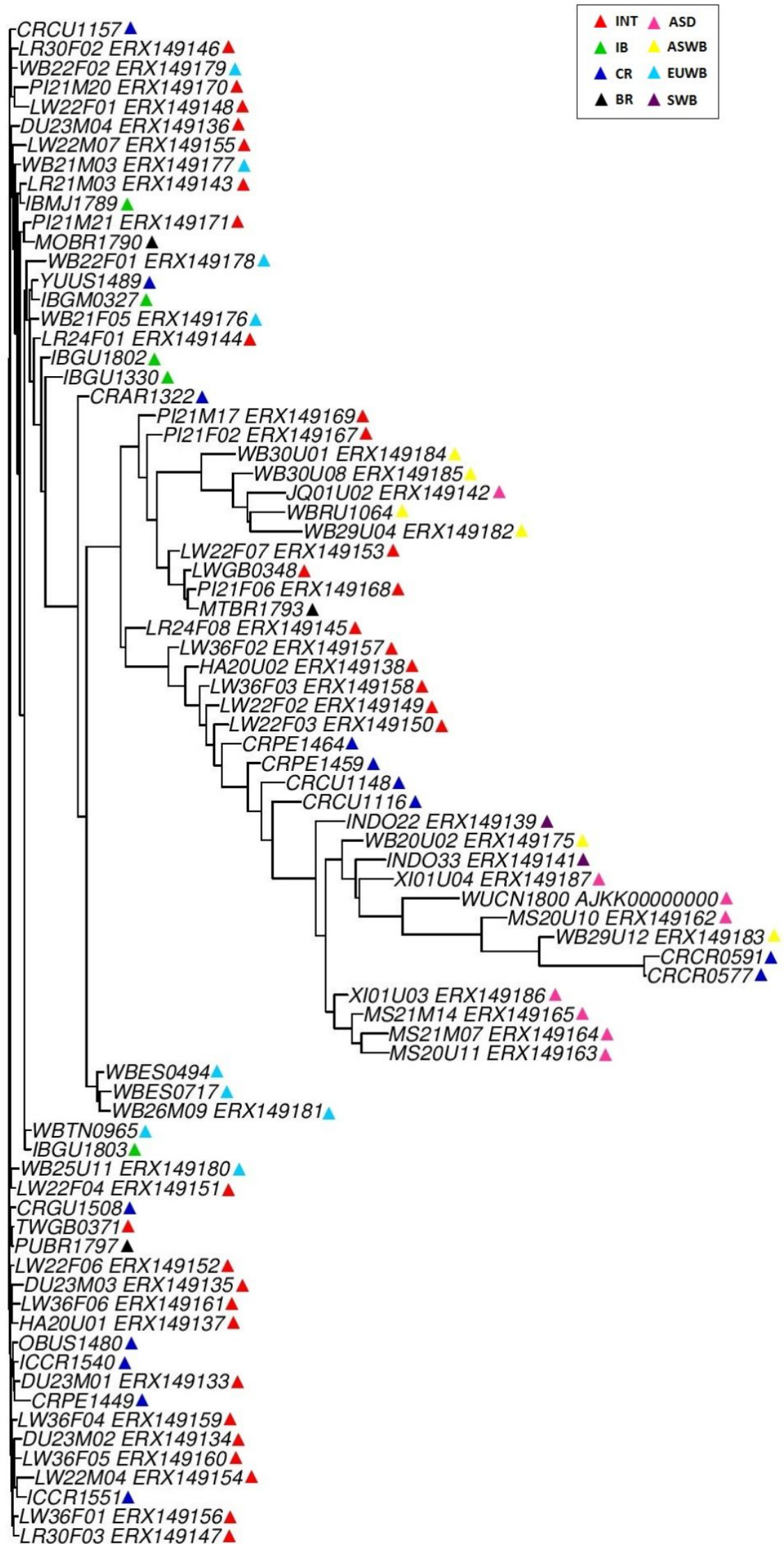

Supplement: Supplementary file 5 — Additional file 5: NJ tree of genetic distances for Tas2R9 gene. Color triangles represent population origins: INT, International; IB, Iberian; CR, Creole; BR, Brazilian; ASD, Asian domestic; ASWB, Asian wild boar; EUWB, European wild boar; SWB, Sumatran wild boar. The first two letters of each sample are the breed code: CR, creole; LR, Landrace; LW, Large White; IB, Iberian; HA, Hampshire; XI, Xian; MS, Meishan; JQ, Jianquahi; TW, Tamworth; DU, Duroc. Note, eg, that six out of 14 LW samples cluster near Asian samples, together with some Creole and Pietrain individuals. (PDF 231 KB) [file 12864_2014_6798_MOESM5_ESM.pdf]
