# Supplementary material for: Characterization of the porcine nutrient and taste receptor gene repertoire in domestic and wild populations across the globe
Source: BMC Genomics. 2014 Dec 3;15(1):1057. doi: 10.1186/1471-2164-15-1057 (PMC4302110; doi:10.1186/1471-2164-15-1057)
Supplement: Supplementary file 6 — Additional file 6: Primer details for the porcine nutrient sensing and taste receptor genes used for estimating relative gene expression levels. (DOCX 19 KB) [file 12864_2014_6798_MOESM6_ESM.docx]

**Additional file 7.** Primer details for the porcine nutrient sensing and taste receptor gene repertoire.

| Gene | Primer | Sequence | Product size (bp) | Gene | Primer | Sequence | Product size (bp) |
| --- | --- | --- | --- | --- | --- | --- | --- |
| *Tas1r1* | FWD | ATCTGTTCTCGAGGCCAAGTCT | 109 | *Tas2r41* | FWD | TTCTCCGAGAGCGACTGGTA | 134 |
|  | REV | GCGAGTCCCCACTGTCACTAA |  |  | REV | CCCCTGGCCAACAGAGTTAG |  |
| *Tas1r2* | FWD | GCCACCATGACCGTGGCCCACTT | 77 | *Tas2r42* | FWD | CTGCCTTTTCCTCTGGCTGA | 300 |
|  | REV | AGGTCCTCACTGATGGCACTGTAGC |  |  | REV | TCCCTCGAGCACACAAAGTT |  |
| *Tas1r3* | FWD | CGCAGCATTGCCACCTACTG | 134 | *Tas2r60* | FWD | TCCTTACATCGCTGGGGAGA | 248 |
|  | REV | TAGCTGACCTGCGGCATGAG |  |  | REV | CAAGAGCAGAACGATGGGGT |  |
| *Tas2r1* | FWD | TTTGGTTTGCCACATGGCTG | 215 | *Tas2r134* | FWD | CTCGTCTTCCACACGTCGTA | 184 |
|  | REV | TGCATTTGGGGACAGAAGGG |  |  | REV | CTCTCCGAAGCCTCTCCTTT |  |
| *Tas2r3* | FWD | GGAATCGGTGGATCAGGCAA | 186 | *O3FAR1/GPR120* | FWD | GATTTGGCCCAGTGTTGCTG | 170 |
|  | REV | GGAGCCGGTAGAGTTTTGCT |  |  | REV | TCTGGTGGCTCTCGGAGTAA |  |
| *Tas2r4* | FWD | GAGGGCATCTTGTCCTTGCT | 160 | *FFAR1/GPR40* | FWD | GCCTAGGACCCTACAATGCC | 187 |
|  | REV | GAGCTTCAGTCTGGGGGTTC |  |  | REV | GATGGGCCCCCTTTTGTTCT |  |
| *Tas2r7* | FWD | TAGGGTGTTTGGCCCTCTCT | 236 | *FFAR2/GPR43* | FWD | GATCCTCACGGCCTACATCC | 201 |
|  | REV | GGACACAGAAAAGGGGAGCA |  |  | REV | TACCAGCGGAAGTTAGACGC |  |
| *Tas2r9* | FWD | ACCGCTAGCTTTCTGATCCC | 213 | *FFAR3/GPR41* | FWD | CCGAGTGGAGACCTTACGTG | 169 |
|  | REV | CTCAGCTTGCTGTTCCCCAT |  |  | REV | TTCTTCAGTTTCCCGCTGCT |  |
| *Tas2r10* | FWD | CAGAGACCCCAGCACAGAAG | 188 | *GPR84* | FWD | CTCATCGCCAACCTCACAGT | 150 |
|  | REV | CCCAAGGAAAAATGGCTGTGG |  |  | REV | GGAGACAGAGTTGGACGCAA |  |
| *Tas2r16* | FWD | GTGACGGAGAGGCTTGAGAC | 116 | *GPRC6A* | FWD | GCCGGGATTTGTCCACAGTA | 160 |
|  | REV | TTTCATCTGCCTCACGTGCT |  |  | REV | TGAGCTCCCTCCATCCATGA |  |
| *Tas2r20* | FWD | CCCAAAATCCCAGCACCAAG | 173 | *GRM1/MGluR1* | FWD | GTCGGGAGCTCTGCTACATC | 230 |
|  | REV | TGAGTGGACTGAAGGCTGGA |  |  | REV | GGCACTCATAAACCTGGGCT |  |
| *Tas2r38* | FWD | CAGCCTGGAGGCCCATTTTA | 138 | *GRM4/mGluR4* | FWD | ATCTTCTTTGGCACCTCGCA | 123 |
|  | REV | AGGCTGCCAGTATCCCTACA |  |  | REV | GACGTAGACCTTGGGCATGT |  |
| *Tas2r39* | FWD | CCTTGGGCCATAGTGGACTC | 182 | *CaSR* | FWD | TGCCCAGATGACTTCTGGTCC | 330 |
|  | REV | CATGTGCTGGGTGTGTCTCT |  |  | REV | GCACGAGATGCAGAGCACGAAGC |  |
| *GPR92* | FWD | TTCCTGCTGTGCTTCGTGCC | 184 | *TBP* | FWD | AACAGTTCAGTAGTTATGAGCCAGA | 153 |
|  | REV | GCCAGCAGCAAGAGTGGCAGCA |  |  | REV | AGATGTTCTCAAACGCTTCG |  |
| *GAPDH* | FWD | TGGTGAAGGTCGGAGTGAAC | 104 |  |  |  |  |
|  | REV | GAAGGGGTCATTGATGGCGA |  |  |  |  |  |
